# How UK health care professionals conceptualise parental experiences of the diagnostic process for autism spectrum disorder: A qualitative study

**DOI:** 10.1177/20503121211031310

**Published:** 2021-07-17

**Authors:** Natasha Faye Daniels, Barry Coughlan, Robbie Duschinsky

**Affiliations:** 1 School of Clinical Medicine, University of Cambridge, Cambridge, UK; 2 Department of Public Health and Primary Care, University of Cambridge, Cambridge, UK

**Keywords:** Autism spectrum disorder, diagnostic pathway, parental experience

## Abstract

**Objectives:**

Much of the literature on diagnostic experiences of autism focuses on parental perspectives. Few studies have explored how health care professionals conceptualise parental experiences of the diagnostic process. The current study examines clinical perspectives of the diagnostic process with a focus on the perceived impact of assessment on families.

**Methods:**

Qualitative interviews were conducted with 25 health care professionals from various National Health Service child and adolescent mental health services and general practices in the United Kingdom. Interviews were transcribed verbatim and data were analysed using a thematic approach.

**Results:**

Two main themes were identified: (1) stress and the autism spectrum disorder diagnostic process and (2) expectations of the diagnostic pathway. The main sources of stress perceived by the health care professionals related to diagnostic delay and ambiguity around the diagnostic process, with parents facing significant hurdles in understanding their child’s behaviour. Many health care professionals also reported a struggle to navigate differing expectations of the diagnostic process between parents and clinicians, as well as managing objectivity in the face of significant distress. Parent internalised stigma and guilt was a key component of the health care professional’s perception of sources of stress around the diagnostic process.

**Conclusion:**

The vast majority of clinicians recognised the diagnostic pathway as a significant source of stress for parents, with many hurdles and battles to finalise the process.

## Introduction

The impact of the autism diagnostic process on the families involved has garnered growing attention. Large survey studies have identified that parents tend to describe the process of obtaining a diagnosis for their child as prolonged,^1,2^ with a reported average delay of 3.5 years between early expressions of concern and formal diagnosis.^1^ In light of these findings, it is unsurprising that satisfaction with the overall diagnostic process is poor.^1^ Indeed, a recurring theme in the literature has been that earlier diagnosis is associated with greater parental satisfaction, with diagnosis during the preschool years yielding the greatest satisfaction.^3^ Qualitative work in the United Kingdom identified that key factors implicated in parental diagnostic dissatisfaction were the inconsistency and ambiguity involved in the diagnostic process.^4^ Crane et al.^4^ identified tension between professionals and parents, with many parents reported entering consultations with a clear diagnosis in mind, which was seen to negatively impact rapport development.

Along similar lines, a recent review by Boshoff and colleagues identified that parents who feel unheard by health professionals may resort to pressuring clinicians for a diagnosis.^5^ Such pressure stems from the fact that parents feel that a diagnosis is required to maximise access to interventions,^6^ which may involve advocacy on behalf of their child. Advocacy is seen as a balancing act between the benefits conferred on their child against their own, and indeed the family unit as a whole. Many parents described the need to advocate for their child through the diagnostic process as exhausting.^5^

There is increasing evidence that early detection of autism can facilitate better outcomes in later life in terms of verbal skills^7^ and developmental gains, with earlier intervention associated with better outcomes.^8^ This idea of an earlier intervention being better can increase the pressure on parents to get their child diagnosed earlier. Somewhat predictably, a delay in the diagnostic pathway correlates to a delay in autism spectrum disorder (ASD)-specific intervention^9^ as an official diagnosis is often a prerequisite for targeted support and entitlements both within the context of the home and school.^10^ With the referral times recommended to be within 3 months^11^ and within 17 weeks for post-referral feedback under the National Autism Plan for Children,^12^ parents are often distressed by the lengthy process.

Transitioning from obtaining a diagnosis to understanding and accepting the diagnosis, and how best to proceed as a family, are ultimately shaped by these waiting times. The legitimacy of ASD as an explanation for both child and parental struggle only comes with a diagnosis, with social recognition and medical confirmation being entwined.^13^ Thus, when the stakes are this high, it is not surprising that parents find the diagnostic process stressful.

Parental anxiety regarding waiting times, if likening autism to other diseases, becomes more understandable if there is the expectation of a complete ‘curative’ alteration in the autistic trajectory. Focus groups with professionals in Scotland suggests that an array of organisational factors, such as high rates of non-attendance, inappropriate referrals and communication difficulties between cognate services are often drawn on to explain delays and increased waiting times.^14^

ASD is behaviourally diagnosed and understanding the experiences of parents is important because parental testimonies are one of the key forms of information used by health care professionals (HCPs) to support referral and diagnostic decisions. For instance, parents are often asked to complete screening checklists, such as the Modified Checklist for Screening Autism in Toddlers^15^ or structured clinical interviews, such as the Autism Diagnostic Interview.^16^

Dumit^13^ points out, one of the qualities of medical encounters concerning conditions with unestablished biomedical aetiology is that there is often a requirement for patients to ‘prove’ their symptoms align with a particular disorder. Yet while patients are attempting to evidence their symptoms professionals are explicitly reminded by professional textbooks^17^ to consider non-medical incentives for subjective symptom-reporting. Furthermore, although the parents are regarded as an authoritative source of knowledge regarding their child, professionals are regarded as the authority on the condition. This creates a complex epistemological terrain for knowledge claims to be disputed and where biomedical evidence does not offer an obvious resolution.

To date, much of the research on parental experiences of ASD assessment has focused on parents. Survey work with professionals indicates that communicating clinical reasoning around diagnostic decisions is one of the most challenging aspects of delivering an ASD diagnosis.^18^ However, to better comprehend this landscape, it is important to consider how professionals understand and reflect on parents’ experiences. Therefore, the current study aims to explore the HCP’s perspective of the parents’ experience of the ASD diagnostic pathway.

## Methods

### Procedure

A qualitative thematic analysis was conducted looking at the perceptions of HCPs regarding the parent experience of the ASD diagnostic pathway. Between January and May 2019, semi-structured interviews were conducted with (*n* = 25) HCPs in the United Kingdom (for details, see Table 1). These included general practitioners (GPs), professionals at local child and adolescent mental health services (CAMHS) and professionals at national specialist social and neurodevelopmental services. Participants were required to have at least 3 years of post-qualification experience. The sample size was determined using Malterud’s Information Power Approach^19^ as opposed to a saturation approach. This approach encompasses the idea that the sample size in qualitative research is dependent on multiple factors, including the aim of the study, sample specificity, use of established theory, quality of dialogue and analysis strategy. This idea of ‘information power’ is used to guide a sufficient sample size, in which the greater the information in a sample, the lower the number of required participants. The participants were recruited via a combination of purposive, snowball and convenience sampling. All interviews were conducted by B.C. and took place either in person or remotely (e.g. by phone or Skype). B.C. has previously worked in a neurodevelopmental service and has experience working with HCPs from cognate disciplines and parents and families of children with autism.

**Table 1.** Professional and demographic characteristics of those interviewed.

Participant ID	Gender	Position	Experience (years)	Interview length (min)
PTGP01	Female	GP	20 +	43
PTGP02	Male	GP	0–5	41
PTGP03	Female	GP	20 +	44
PTGP04	Male	GP	20 +	64
PTGP05	Male	GP	16–20	29
PTGP06	Male	GP	20 +	37
PTGP07	Male	GP	20 +	71
PTGP08	Male	GP	11–15	61
PTND01	Male	Paediatrician	16–20	66
PTND02	Female	Allied health professional	20 +	64
PTND03	Female	Allied health professional	11–15	58
PTND04	Female	Paediatrician	20 +	64
PTND05	Female	Psychologist	11–15	69
PTND06	Female	Psychologist	11–15	65
PTND07	Male	Psychologist	0–5	55
PTND08	Female	Psychologist	6–10	62
PTND09	Female	Psychologist	16–20	53
PTND10	Female	Allied health professional	6–10	58
PTND11	Female	Psychologist	16–20	48
PTND12	Male	Psychologist	6–10	54
PTND13	Female	Psychologist	20 +	43
PTND14	Male	Psychologist	20 +	55
PTND15	Female	Psychiatrist	0–5	61
PTND16	Male	Psychologist	0–5	63
PTND17	Female	Psychiatrist	20 +	65

GP: general practitioner.

Prior to data collection, a flexible topic guide was prepared and piloted with three psychologists. Topics and questions were then conferenced with two academic GPs to confirm relevance to general practice. A patient and public involvement panel at a local hospital provided additional feedback on the interview materials which resulted in a bespoke interview guide for the qualitative research. After piloting a proposed symptom matching task was abandoned to allow greater time for participants to reflect on routine cases. Minor adjustments were also made to ensure the tone of the questions was aligned with the aims to explore perspectives rather than evaluate knowledge. The final interview schedule contained the following topics: (1) professional background, (2) routine clinical work, (3) hypothetical case study and (4) referral pathways (see Supplemental Appendix). Ethics and governance approvals were provided by a University of Cambridge Psychology Committee (PRE.2018.019), health research authority, and relevant National Health Service (NHS) research and development teams. All interviews were audio-recorded and transcribed verbatim. Participants provided written and verbal consent prior to the interview. Verbal consent was also confirmed at the end of the interview. At the beginning of each interview, participants were asked not to reveal any personally identifiable information about children or families in their services. Participants were also reminded of this prior to discussions of routine clinical work.

These interviews were conducted as part of a multi-pronged doctoral research project on social and neurodevelopmental assessment in children. Findings regarding assessment practices, referral pathways and differential diagnosis are presented elsewhere.^20–22^ The findings presented in the current article focus exclusively on participants understanding of the experiences of parents.

### Analysis

The data were analysed using the thematic approach outlined in the work of Braun and Clarke.^23^ N.F.D. and B.C. read each of the transcripts twice prior to coding to become familiarised with the data. Data were coded by N.F.D. using a line-by-line approach with codes then grouped into descriptive and analytical themes (Table 2), with consensus achieved with B.C. after discussion of each transcript. The research group held regular meetings to discuss these themes and develop analytical themes. Data were organised using Microsoft word and NVivo 12. Frequent meetings and discussions between researchers were conducted to share opinions with the anticipation of minimising and challenging any apparent individual bias. The analysis was predominantly deductive in nature. While this was not originally envisaged, the authors were struck by how close the participants’ remarks floated around pertinent topics in the literature.

**Table 2.** Themes and their constituent subthemes after qualitative analyses.

Themes	Subthemes
Stress and the ASD diagnostic pathway	1. Delayed diagnosis as a significant source of stress for parents 2. Parental battle with services
Expectations of the diagnostic pathway	1. Parental determination and clinician objectivity 2. Recognition of parental internalised stigma and guilt

ASD: autism spectrum disorder.

### Critical realism

The approach in this study was informed by critical realism. Therefore, it was contended that there is a material difference between ontology and epistemology. To varying degrees, each author holds the view that autism is a real phenomenon,^24^ but our knowledge about ASD is invariably linked to a variety of factors including interest groups, assessment practices, policies and dominant research paradigms. Therefore, the work is less oriented by the concern of whether ASD is real as such, but rather how this concept is applied and whether it is helpful.

### Reflexivity

B.C. has a keen research interest in ASD assessment practices and has previously worked in a service for children with neurodevelopmental difficulties. He has worked with several parents and professionals involved in the assessment process, and he is interested in how knowledge claims and territories are negotiated in practice. N.F.D. has a personal interest in the ASD diagnostic process and has worked with charities involved in the support of children with ASD. She is particularly interested in how parents navigate the diagnostic process and the personal impact on them.

## Data collection

Table 1 shows the professional and demographic characteristics of those interviewed.

## Results

### THEME: stress and the ASD diagnostic pathway

#### Subtheme 1: delayed diagnosis as a significant source of stress for parents

The biggest concern cited by clinicians regarding the parent experience of the diagnostic process was the long wait to see a specialist to determine whether there was a formal diagnosis of ASD. This was seen as a significant source of stress both in terms of the uncertainty such a delay held for parents, and the rush to receive intervention due to a perceived time frame of efficacy:

Families are given intimation that something might be amiss and they get all these messages in the media that early intervention is key. And then they basically have to wait which I’m sure is very very challenging. (PTND07)

I think the waiting list in the UK is at least two years in most parts of the country two years plus in some parts. So I think most families’ experience is quite negative. (PTND11)

Really badly. So I guess in different places I’ve worked with feedback from families is that it’s often a very slow process. (PTND12)

It is clear that there is concern among clinicians on behalf of the families about delayed diagnosis, and that many clinicians understand that this is a source of stress for families. There was a sense that such delays were due to organisational shortcomings and signalled a tension with the professional’s own values. One clinician suggested that a long wait would be ‘bad practice’, without acknowledging that this appears to be what many families experience in reality. It seems there is incongruency between what clinicians want for their patients and families and what they perceive as general trends in service provision.

Interestingly, in one local service, however, where some of the participants had been involved in organising referral pathways, clinicians felt that this had become less of a problem:

What we’re seeing now is actually no worries about the length of waiting times. Actually feeling that it’s moved through quite quickly so generally, I think the experience is good. (PTND02)

Although the parents are anxious when they think there’s a problem . . . they always think it [diagnosis] takes too long. (PTND04)

#### Subtheme 2: parental battle with services

In several transcripts, clinicians recognised that parents face barriers when seeking a diagnosis of ASD, both in the school environment and health service. The language that clinicians used to describe these barriers was typically emotional in nature. The use of words, such as ‘fight’ and ‘resilient’ indicate a struggle for the parents to get the support they need in the diagnostic pathway. The frequency with which emotive words, such as ‘terrible’ and ‘frustrating’ permeate many of the transcripts suggests an undercurrent of frustration at the system for the clinicians. This likely speaks to perceived tensions between commissioners and frontline staff. The fact that it makes certain clinicians ‘a bit cross’ and that the clinicians themselves must ‘fight’ with cognate services reflects a struggle throughout the diagnostic pathway, both for providers and those seeking care:

So I think it makes me a bit cross because I think it’s [the diagnostic process] very confusing for parents and carers. (PTND02)

But parents may have found out about girls on the spectrum read about it and pursue getting a diagnosis but they have to be very resilient . . . have an absolutely terrible time. (PTND09)

Because of the restriction of services especially young families they fight for it. (PTND06)

I think they find it an extremely long arduous and frustrating battle and that only with great persistence and endurance do they get somewhere like here. (PTND09)

The use of military language, such as ‘fight’, ‘resilient’, ‘battle’ and ‘endurance’ suggest that clinicians recognise a substantial struggle by parents for an ASD diagnosis. This poignant language indicates that clinicians have sympathy for what they see as a barrier-laden process. Interestingly, there is marked variation in the intensity of the language used, from ‘some concerns’ and ‘a bit cross’ to language, such as ‘frustrating battle’ and ‘absolutely terrible time’. This suggests a varying degree of perceived struggle in terms of parental experience.

### THEME: expectations of the diagnostic pathway

#### Subtheme 1: parental determination and clinician objectivity

An important element to the diagnosis of autism is the parental perception of atypical development, as it is often the parents who first contact medical professionals regarding developmental concerns.^4^ At times, there was a clash perceived between the need for clinicians to consider a diagnosis objectively and dispassionately based on medical evidence, and the parental determination to get the diagnosis they believed to be correct. In some cases, specific parental motives were perceived to underlie this determination to get an ASD diagnosis. It appears that of the clinicians who chose to comment on a parental motive, there were two main hypotheses offered for this:

1. Parents wanting a diagnosis to offload responsibility for their child’s behaviour.
2. Parents wanting a diagnosis to access benefits.

While both are legitimate reasons for wanting a diagnosis, the clinicians’ reactions to them were negative, likely due to the perceived ‘pressure to perform’. Maximising an objective stance when you have a distressed parent with a very clear need for a diagnosis is understandably difficult. Balancing such objectivity with the goal of beneficence undoubtedly increases the pressure felt to make a diagnosis:

[The] parent was giving a very clear consistent developmental history that’s in line with Social Communication Disorder . . . some old reports that we had seen that were less consistent so I think that made us concerned about the reliability of the parent history and also the motivation behind the parent giving the history in that way. (PTND08)

They come very invested and wanting to have a diagnosis to explain everything away. (PTND02)

So for instance, a mother wants a diagnosis because of benefits. (PTND04)

If there’s a vested interest in having a diagnosis, people can very easily overestimate [the symptom burden], so I don’t think it’s useful in and of itself. (PTND07)

Are parents so knowledgeable about autism, or [do they] really feel that they need this diagnosis? Are they answering the questions? And maybe it’s biasing the interview but at the same time we did feel he was a bit unusual. (PTND09)

Some of them [referrals] are basically done based on kind of genuine concern. Some of them are done mostly because the parents have been pushing for a lot. (PTND16)

Relieving the guilt of being a ‘bad parent’ was suggested as a means of shifting parental responsibility rather than a form of emotional relief for the parents. There is the recurrent use of the phrases ‘invested’ and ‘vested interest’ which could reflect scepticism regarding parents attempting to understand their child’s difficulties. Understandably when having to make an objective diagnosis, the added pressure from parents with a clear outcome in mind can make such an objective journey difficult.

Furthering this perceived scepticism is one specialist clinician describing the situation as follows:

Mum was quite hooked on this must be autism. (PTND02)

Similar language was observed in another transcript; ‘they were convinced he’s got a diagnosis of autism’. The use of the word ‘convinced’ and ‘hooked on’, like ‘invested’, signals scepticism of the parent’s judgement, or at least perceived parental inflation about the child’s presenting differences. Interestingly, such notions featured more in specialist clinicians rather than GPs, and those with more experience demonstrated a greater degree of scepticism around parental perceptions.

While there have been many examples of divergent thinking between parents and clinicians, there were also cases recounting convergent thinking and teamwork between parents and clinicians:

Mum actually said at the end can I give you a big hug? It was really lovely so what I’d anticipated going into thinking this is going to be really difficult, cos I’m going to say it’s not ADHD and this mum’s going to be really cross and actually it was completely opposite she’d had two weeks to go away and think about this and talk with her family and think about those things. (PTND02)

Just got to go with what the parents are thinking. (PTGP06)

as I say to parents you know your son or daughter better than anybody in the world. So we have to listen to what they have to say ideas concerns and expectations. (PTGP07)

Just occasionally will say to parent well this is a bit borderline. Well. It’s just about ASD. We’ll almost ask them which way they want us to jump. (PTND16)

#### Subtheme 2: recognition of parental internalised stigma and guilt

The concept of parental guilt pervaded many of the clinician’s accounts of the parental experience, with ‘blame’, ‘defensive’ and ‘criticise’ recurring words with extremely powerful connotations. These terms suggest that the parent is possibly sensitive to the potential for judgement, and appears to be a limiting factor in honest discourse. The concept of blame in several interviews suggests this may be a focal reason underpinning hesitancy in accepting help regarding parental style. Examples emphasising the theme are shown below:

Though they were very brave to accept some support. (PTND05)

They feel a neurodevelopmental diagnosis validates them and exonerates the parents it’s got nothing to do with the upbringing and those kind of things. (PTND01)

parents already thinking we’re blaming them and constantly reassuring them that we’re trying to find an explanation not blame. (PTND01)

very sad and tearful. I talked with her very much about look this isn’t about blame

Very often parents would feel blamed. (PTND16)

It’s just thinking about how to address with parents the issue so they don’t feel blamed. Or being ‘bad parents’. (PTND16)

The idea that a parent is ‘brave’ in accepting help indicates that there is an element of vulnerability when allowing ones parental style to be analysed, especially in light of the historical blame that society puts on parents regarding ASD and the power of the clinician to assign such blame:

you get a cohort of children who can present as though they have ADHD where actually the attention deficit they have had is from their parents. (PTGP04)

The concept of emotionally unavailable mothers being responsible for the autistic child’s ‘withdrawal’ from society^25^ coupled with the fact that ASD remains poorly understood in the public discourse explains such reticence by the parents. Such hesitancy is not surprising in light of a system that demands a label to legitimise resource allocation, while making such a label notoriously laborious to obtain.

Although a formal discourse analysis was not performed, elements of critical discourse analysis were used to explore how discourses are employed to navigate different knowledge territories between parents and clinicians. Here, we see three HCPs offer reflections on instances when there was a misalignment of parental and clinical conceptualisations of the presenting difficulties:

We get quite a lot of complaints from parents. Usually because we haven’t given a diagnosis of something they’ve decided the child has. (PTND17)

what we tend to call the Something Must Be Done scenario where you think actually this lad is the product of his genetics. (PTGP04)

we had seen that were less consistent so I think that made us concerned about the reliability of the parent history. (PTND08)

There are several noteworthy features of these quotations. First, the use of the word ‘we’ indicates a shared issue around managing the ASD diagnostic pathway. This suggests that the situations described are not unusual or unique, as ‘we’ generalises it across the medical profession. The tensions between parents and clinicians are centred around ‘we’ and, thus, there appears to be a collective issue around how HCPs perceive parent concerns.

Furthermore, to varying degrees in each of these quotations, the parents’ role as ‘reliable narrator’ of the child’s difficulties is thrown into doubt. This is of course not to say that there are not legitimate reasons for disagreements between parents and clinicians. Still, there is a noteworthy asymmetry of power here in relation to parents and the recourse they have when they disagree with diagnostic decisions.

Indeed, much of the language used indicates a struggle for power and authority, or hegemony as described by Fairclough (2001):^26^

grandmother seemed to know it all. But they weren’t having that because they knew it all. And they’d done some research and were thinking it was ADHD. It sounded a bit more complex than that to me. (PTGP05)

issues to do with power such as title of patient and the child has a problem and as us as clinicians trying not to see it that way. And to convey to the family that it is not the case. (PTND06)

Are parents so knowledgeable about autism, or [do they] really feel that they need this diagnosis. (PTND09)

The language used, such as ‘so knowledgeable’ and ‘know it all’ indicate a convention in medicine where patients describe symptoms and HCPs provide diagnoses. This discourse helps to define and reinforce two distinct but overlapping knowledge territories. That is, within this interaction, parents are the authority on their child, but clinicians are the authority on ASD.

## Discussion

Through interviewing professionals involved in the autism diagnostic process and integrating their insights into the process, a set of themes were identified. The two key themes centred around the diagnostic process as a key source of parental stress, disparities between the parent and clinician experience of the pathway and variations in the clinical and parental understanding of ASD behaviours. Perhaps, the most prominent theme was the concept of parental stress and anxiety being directly related to the length of time to diagnosis, something many of the clinicians themselves were unhappy with.

National Institute for Health and Care Excellence (NICE) guidance currently recommends a referral time of 3 months to diagnostic assessment^11^ with the National Autism Plan for Children (NAPC) recommending no greater than 17 weeks for post-referral feedback.^12^ *PTND11* and *PTND12* recognise that in some areas, the waiting times can exceed 2 years, highlighting that stretched services are unable to meet these guidelines, which is concordant with Public Health England data showing that only 22% of local authorities met the 3-month target.^27^ The vast majority of clinicians interviewed expressed sympathy for the parents in their struggle to be seen, and they were not happy with the waiting times. Longer diagnostic waiting times are associated with parents having reduced confidence in their medical contact;^28^ which could have implications for future encounters. Parental satisfaction with the overall diagnostic experience has been found to be very dependent on timely diagnosis,^4^ with satisfaction being greatest when a diagnosis was received in the preschool years.

The vast majority of clinicians interviewed demonstrated sympathy towards the parents around waiting times and seemed equally frustrated by the slow movement through the pathway. Few participants highlighted that their services managed to ensure that ‘no corners are cut’ while having waitlists below the national averages. Still, there was an acknowledgement that these waiting times were atypical. It is important to note that with the general population having a greater awareness of ASD and its manifestations via mass media and personal contacts,^29^ expectations for a quick and comprehensive service are likely to be elevated which could skew perceptions of waiting times.

As discussed in the literature review, the importance of garnering clinicians’ perspectives of the parent experience lies in the provision of a different vantage point on perceptions of collaborative case-conceptualisation. As much of the qualitative research is directly based on parent experiences, getting the viewpoints of HCP’s aids in seeing how much of this experience filters into the HCP’s perception of the diagnostic process. Indeed, looking for discrepancies could aid in educating HCPs about the struggles of parents. Reassuringly, this article highlights that clinicians generally view parents as providers of symptom information, and their importance in the overall process of formulating accurate diagnoses. Moreover, this research aims to narrow the gap in experience between parents and clinicians by highlighting the differences, in the context of existing literature around parental experiences.

It is common for parents to be the first to detect developmental variations with their child.^4^ Despite this, there was a reticence for some clinicians in the study to accept parental observations. Arguably, the balance between the expertise of parents and professionals is a difficult one to strike, with both having very different forms of experience and knowledge regarding ASD behaviours. It is clear that some of the professionals interviewed were frustrated by the parent entering the consultation with a specific diagnosis in mind; which can overlook other potential diagnoses. The role of the clinician is to consider all possibilities before making a final diagnosis, thus, it is understandable that the professionals may become frustrated by perceived pressure to ignore other options. Nevertheless, the language (e.g. ‘hooked on’) used by a minority of the clinicians’ risks delegitimising parents’ concerns. Still, it is important to state that questioning motives for a diagnosis are not atypical practice. In fact, explicit recommendations to do so can be found in authoritative textbooks for professionals.^17^ Yet what is often lacking is guidance about how this should be done.

There was a perceived clash between the objective knowledge and clinical training of the professional, and the subjective experience of the parent. The epistemic authority granted to the clinician has somewhat withered with time, especially with the rise of alternative medicine.^30^ This makes the reticence of the clinicians to forgo their authority understandable; in a way, it can be seen as a protective mechanism for patients against misinformation. While some clinicians interviewed recognised a stubbornness on part of the parent determined to get the diagnosis with language like ‘fixed’, in the majority of cases clinicians considered the parent as a key source of useful information; striking that balance of epistemic values. Involving autistic people in the configuration of services could aid the development of a balanced partnership between clinician and patient, and the gulf of differing experiences to be channelled into a more constructive consultation.

It is important to note some limitations of this study. First, as this is not an observational study the authors cannot comment on how clinicians are in their clinical practice, relying solely on their perceptions of their practice. Second, as the sample included professionals at specialist services, they are potentially more likely to encounter complex cases that are not necessarily reflective of the general patient population. As only the views of the HCPs have been taken and not of the parents of whom they speak, there may be bias in favour of clinicians. In addition, it is important to note the role of salience in the results. The goal was to provide an account of how clinicians conceptualise parents’ experiences of ASD assessment and drawing on a range of diagnostic encounters in different services. Part of the interview asked clinicians to reflect on attachment -related cases and uncertain cases. Therefore, that might have prompted clinicians to discuss more complex cases and reflect on less routine cases. Furthermore, some of the clinicians worked at expert services.

Parents can feel isolated due to a perceived societal lack of understanding of the nature of autism, in addition to the parental struggles involved in caring for a child with autism.^31,32^ This lack of recognition fuels the stigma felt by parents. The judgement is particularly felt in times of public tantrums, where parents may feel guilty due to people staring or blaming ‘naughtiness’ for the behaviour rather than ASD.^32,33^ The stigma felt by parents is twofold: they can be blamed for the ASD itself, and also for failing to change or control such resulting behaviours. It is no surprise that such repeated felt stigma eventually manifests into self-blame in many parents of children with atypical behaviours.^34^ Within this context, therefore, it might be beneficial to raise awareness of these struggles within the health care community to better understand some of the tensions perceived by the HCPs.

In addition to the emotional impact of diagnostic delay for the parents, evidence suggests that long waiting lists are more associated with parents seeking out substitute treatments for which there is little empirical support.^28^ This might increase the risk of parental desperation being exploited, with data suggesting that the uptake of complementary and alternative medicine (CAM) in children with ASD is higher than in the general population and in other psychiatric conditions.^35^

## Conclusion

The clinicians’ appraisal of the parental experience of the diagnostic process varied, however, the vast majority of those interviewed conceded that the waiting times were a significant source of stress and anxiety for parents and themselves were concerned by it. The concept of guilt among the parent population was seen in a significant number of clinicians’ responses, with a sense of reticence experienced when considering parental strategies as a line of questioning. The majority of clinicians were sympathetic to what they perceived as an intensely stressful process for parents, implying that the current process is not considered acceptable, by both parents and health professionals. This article highlights that both parents and experienced clinicians perceive the current diagnostic process as unsatisfactory; thus, a revision of the current process to incorporate these perceptions is important. At a minimum, raising awareness of the stress felt by both parents and clinicians is essential to ensure the provision of adequate emotional support. This research highlights the need for greater support for parents before a formal diagnosis is received, and more guidance for parents to bolster understanding of the diagnostic pathway and aid expectations.

## Supplemental Material

sj-pdf-1-smo-10.1177_20503121211031310 – Supplemental material for How UK health care professionals conceptualise parental experiences of the diagnostic process for autism spectrum disorder: A qualitative study Click here for additional data file. Supplemental material, sj-pdf-1-smo-10.1177_20503121211031310 for How UK health care professionals conceptualise parental experiences of the diagnostic process for autism spectrum disorder: A qualitative study by Natasha Faye Daniels, Barry Coughlan and Robbie Duschinsky in SAGE Open Medicine
