# Supplementary material for: How UK health care professionals conceptualise parental experiences of the diagnostic process for autism spectrum disorder: A qualitative study
Source: SAGE Open Med. 2021 Jul 17;9:20503121211031310. doi: 10.1177/20503121211031310 (PMC8287381; doi:10.1177/20503121211031310)
Supplement: sj-pdf-1-smo-10.1177_20503121211031310 – Supplemental material for How UK health care professionals conceptualise parental experiences of the diagnostic process for autism spectrum disorder: A qualitative study [file sj-pdf-1-smo-10.1177_20503121211031310.pdf]

UNIVERSITY OF CAMBRIDGE
Department of Public Health and Primary Care

NHS
Cambridgeshire and Peterborough
NHS Foundation Trust

Interview Schedule

Introduction:

I would like to ask you some questions about your training and background, your routine clinical activities, and your experiences and views on the diagnostic process for developmental conditions in children. I would like to remind you not to disclose any personally identifiable information about any individual during the interview.

Background

1. Can you give me a brief overview of your current position?
   Probe for experience related to child development

2. How long have you been working as a (GP, psychologist, psychiatrist etc)?
   Probe for length of time working with children and families

3. Where and when did you do your clinical training?
   Probe for General Practitioner (GP) training vs medical training

Differential diagnosis

1. Without revealing any personally identifiable information, can you tell me about a case where you conceptualised the child's features as relating to autism?
   Probe action steps
   Probe specific difficulties with working with this child
   Probe for informal assessment (e.g. familiarity with things in the past)
   Probe assessments (I know X is saturated with screening tools/assessments; observations)
   Probe for level of clarity (how certain or uncertain were you about X)
   Repeat for ADHD and attachment related

2. If not clear, can you give me an overview of your involvement in relation to identification and assessment of developmental (or behavioural) difficulties?
   Probe for assessment tools, observations and focus on developmental histories, team-based approach etc.
   If GP, probe for screening tools.

3. Can you think of a specific instance where there was uncertainty regarding the nature of a child's difficulties/diagnosis?
   Probe for description
   Probe for action steps

4. Are there other factors that contributed/routinely contribute to the decision you made (e.g. system factors, availability of supports and services)?

5. Have you ever been involved with overturning or changing a child's diagnosis?

Clinical Case Study

Now I will ask you about your thoughts on a hypothetical case study. Please take as long as you need to read the case and you will have the case to review when we discuss. If GP, then hand participant case study one. If psychologist, psychiatrist, or allied health professional, hand participant case study two

Case Study 1:
Reception received a phone call from patients Linda (33) and Tim (32) regarding an appointment for their son, Robert (6). The family are known to the practice and previously there have been safeguarding concerns and social services have been involved with the family.

In the initial phone call, Linda requested the next available appointment with the GP. They were subsequently booked in for an appointment in two weeks' time. The next day, Tim phoned reception to express his dissatisfaction with the waiting list and requested that they be given priority in the event of a cancellation.

Two weeks later Linda, Tim, and Robert arrived for the appointment. From the outset, Robert appeared distressed (i.e. crying). Linda made numerous attempts to comfort Robert, but he moved away in response to each of her approaches. At one-point Robert kicked out at Linda. There are what look like two distinctive episodes of hand-flapping.

As the consultation progressed, Robert gradually became more comfortable and was very active (e.g. jumping around the room). He moved from one activity to another in quick succession. Robert's eye contact was fleeting, and seemed to have a restricted range of facial expressions. In terms of conversation, Robert spoke in complex sentences, although the subject matter was a little repetitive and mainly around his favourite toy (Shopkins). Tim then took Robert to the waiting room, so Linda could discuss their concerns with the GP.

According to Linda, Robert has few friends in school and teachers are concerned about his academic progress. Additionally, Robert has become increasingly aggressive towards her and recently threw her laptop at a wall. In terms of history, she reports no significant issues with birth or pregnancy. Robert achieved his motor milestones; however, his language development was delayed. Previously, he received speech and language therapy in the community. Robert has an older half sibling, Chris (14) who has a diagnosis of ADHD. When asked about development prior to three years, Linda disclosed that Robert lived with his grandmother beginning when he was 18 months old to just after his third birthday, as Linda and Tim were separated during this period. During the separation, Linda was an inpatient at a local mental health facility.

Case Study 2:
Robert (aged 6) was referred to the CAMHs service by his general practitioner due to concerns regarding his behaviour and overall development. Robert has made little progress, academically, since starting school one year ago and is described as having few friends. In addition, he has been growing increasingly aggressive towards his mother, Linda (33), and recently threw her laptop computer at a wall. According to Linda, his behavioural issues are longstanding, and she is finding it difficult to manage. Previously there have been safeguarding concerns and social work has been involved with the family.

A developmental history questionnaire revealed no significant issues with pregnancy or birth. Under the strengths section of the questionnaire, “none” was written. Robert achieved his motor milestones; however, his language development was delayed. Previously he received speech and language therapy in the community. The questionnaire also revealed that Robert has an older half sibling, Chris (14) who has a diagnosis of ADHD. The referral was accepted on the basis of the paper screen.

In the first instance, Robert was booked in for an unstructured behavioural/play assessment and offered a place on a parenting programme. Prior to the assessment, the team received a number of phone calls from Robert’s father, Tim (32). In the first phone call Tim said he would not attend the parenting course as parenting isn’t the issue and that he found the invitation insulting.

Robert was on the waiting list for just over 3 months. Over this period, Tim phoned the team a number of times to express his dissatisfaction with the waiting list. At one-point Linda phoned crying saying Robert was “out of control”.

One month later Robert attended the assessment. From the outset, Robert appeared distressed (i.e. crying). Linda made numerous attempts to comfort Robert, but he moved away in response to each of her approaches. At one-point Robert kicked out at Linda. There are what look like two distinctive episodes of hand-flapping.

During the assessment Robert presents as active (e.g. jumping up and down, throwing ball in the air) and moved from one activity to another in quick succession. Robert’s eye contact was fleeting, and he did not demonstrate the full range of facial expressions. In terms of conversation, Robert spoke in complex sentences, although the subject matter was a little repetitive and mainly around his favourite toy (Shopkins).

After the assessment, Tim and Linda completed the autism diagnostic interview (ADIR). The assessment lasted 2hrs 30 mins. When asked about development prior to three years, Linda disclosed that Robert lived with his grandmother beginning when he was 18 months old to just after his third birthday, as Linda and Tim were separated during this period. During the separation, Linda was an inpatient at a local mental health facility. Robert’s score on the ADI-R was just below the threshold for autism.

Case Study Questions

Questions for participants with Case Study 1,

1. What are your initial thoughts on the case?
2. What questions would you ask A) Robert B) Parents (Tim & Linda)
3. What would you advise as the next steps?
4. If the participant deems onward referral is necessary
   a) what information would you put in the referral letter?
   b) Why did X resonate with you?
   c) How easy have you found it to get external help

5. What did you find yourself drawing on to make sense of Roberts presenting difficulties? (i.e. other similar cases, diagnostic criteria, theory etc)

6. If the child had a private diagnosis of autism, would that influence how you think about the case?

7. If you were unsure about the nature of the child's difficulties, are there other professionals you would consult with?

Questions for participants with Case study 2.

1. What are your initial thoughts on the case?

Probe for possible diagnosis, working clinical hypotheses, formulation

If the participant states a specific diagnosis/conceptualisation

A. What features of the case helped you to arrive at that conclusion?
B. Are there other conditions you considered?
   Probe, if so what?
   Probe how the participant differentiated

If the participant indicates that it could be multiple conditions

A. You mentioned that it could be X or Y, how would you differentiate?
B. What assessments/sources of information would be useful in helping you to reach your decision?

If the participant indicates there isn't enough information to make the decision

A. What information would help you make a decision regarding the nature of the child's difficulties?
B. Are there specific assessments/ theories you would use?

2. In your view, is there need for further assessment?
   If Yes, what are the next steps in terms of assessment?

Probe for risk assessment
Probe cognitive assessment
Probe sources of information

3. Are there any frameworks/ theories you used to understand the nature of the child's presenting difficulties?
   Probe for specific models/ theories
   Probe for influential book/text/ or talks

4. If the child had a private diagnosis of autism, would that influence how you think about the case?

5. If you were unsure about the nature of the child's difficulties, are there other professionals you would consult with?

Referral Pathways Information

1. In your experience, what do you consider the early markers of autism?
   Probe for symptoms, features, predisposing events, comorbid conditions, family history
   **repeat for ADHD, Attachment related difficulties.

Rotate order each interview

Questions for Primary Care Clinicians

A. Have you ever referred a child to a CAMHS or relevant assessment service as you suspected the child may a developmental condition or behavioural difficulty?
B. If yes, what information did you include in your referral letter?
C. Have you ever had a referral of this nature not accepted in the first instance?
D. If yes, why was the referral not accepted?
E. On a scale of 1-10, how easy have you found it to get formal assessment for a child you think refer a child who you think may have autism?
   Repeat for attachment problems
   Repeat for ADHD
F. Have you experienced any challenges to referring a child who you think may have autism?
   Repeat for attachment problems
   Repeat for ADHD
G. What, if anything could be done to make the referral pathway, clearer?
H. On a scale of 1-10, how confident do you feel that you will be able to get the appropriate a) assessment b) support for a child with autism, adhd, attachment problems.

Questions for non-primary care clinicians

A. Do you receive many referrals to your service from GPs?
B. If yes, how would you describe the quality of these referrals?
   Probe what's in a good/less good referral
   If no, where do you get the majority of referrals from
   How would you describe the quality of these referrals?
C. What information would constitute a sufficient referral (i.e. allows you to make your decision regarding acceptance for subsequent assessment/support)
D. What information could be collected by a GP to assist with the decision to accept or reject a referral to your service?
E. How do you think children and families experience the journey from primary care to your service?
F. Thinking about conditions with overlapping features, do you see value in differentiating autism from attachment-related conditions?
G. Do you see a meaningful distinction between the attachment disorders (RAD and Disinhibited) and attachment-related difficulties?

H. Similarly, when considering a diagnosis of autism and adhd, what value do you see in diagnosing both or trying to differentiate?
I. Finally, what in you view is the value in differentiating ADHD from attachment-related conditions?
